# Label-free third harmonic generation imaging and quantification of lipid droplets in live filamentous fungi

**DOI:** 10.1038/s41598-022-23502-4

**Published:** 2022-11-05

**Authors:** Tanja Pajić, Nataša V. Todorović, Miroslav Živić, Stanko N. Nikolić, Mihailo D. Rabasović, Andrew H. A. Clayton, Aleksandar J. Krmpot

**Affiliations:** 1 Faculty of Biology, Institute of Physiology and Biochemistry, University of Belgrade, Studentski trg 16, Belgrade, 11158 Serbia; 2 Institute for Biological Research “Siniša Stanković”, University of Belgrade, National Institute of the Republic of Serbia, Bulevar Despota Stefana 142, Belgrade, 11000 Serbia; 3 Institute of Physics Belgrade, University of Belgrade, National Institute of the Republic of Serbia, Pregrevica 118, Belgrade, 11080 Serbia; 4 Department of Physics and Astronomy, Optical Sciences Centre, School of Science, Computing and Engineering Technologies, Swinburne University of Technology, Melbourne, VIC 3122 Australia

**Keywords:** Biophysics, Biological fluorescence, Multiphoton microscopy, Biophotonics, Biological physics, Nonlinear optics, Imaging techniques, Microscopy, Multiphoton microscopy, Imaging studies, Lipids, Lipids, Fluorescence imaging, Optical imaging, Imaging, Microscopy, Multiphoton microscopy, Cellular imaging, Organelles, Fungal physiology, Fungi, Fungal biology, Fat metabolism

## Abstract

We report the utilization of Third-Harmonic Generation microscopy for label-free live cell imaging of lipid droplets in the hypha of filamentous fungus *Phycomyces blakesleeanus*. THG microscopy images showed bright spherical features dispersed throughout the hypha cytoplasm in control conditions and a transient increase in the number of bright features after complete nitrogen starvation. Colocalization analysis of THG and lipid-counterstained images disclosed that the cytoplasmic particles were lipid droplets. Particle Size Analysis and Image Correlation Spectroscopy were used to quantify the number density and size of lipid droplets. The two analysis methods both revealed an increase from 16 × 10^−3^ to 23 × 10^−3^ lipid droplets/µm^2^ after nitrogen starvation and a decrease in the average size of the droplets (range: 0.5–0.8 µm diameter). In conclusion, THG imaging, followed by PSA and ICS, can be reliably used for filamentous fungi for the in vivo quantification of lipid droplets without the need for labeling and/or fixation. In addition, it has been demonstrated that ICS is suitable for THG microscopy.

## Introduction

Third harmonic generation (THG) microscopy as a label-free nonlinear imaging technique is a powerful tool for visualization of various cells and tissue structures^1^. THG has been mainly applied to imaging animal cell structures^1–7^ and tissues^1,4,6,8–14^, as well as the dynamics of cellular processes (functional imaging)^1,6,12,15^. Also, THG microscopy has been used to study human and fossil vertebrate teeth^16^, 3D engineered human adipose tissue^17^, and small organisms (Drosophila melanogaster, zebrafish, Xenopus laevis, early mouse embryos^8,18–20^ and C. elegans^21,22^). In addition to animal specimens, THG microscopy has also been applied to plants^11,23–27^, algae^26,27^ and yeast^2,28^. To the best of our knowledge, there is a paucity of THG studies on filamentous fungi.

The THG phenomenon is a nonlinear coherent scattering process induced by structures with specific properties. In THG, the joint energy of three photons is converted into one photon. As THG is a third-order process, ultra-short laser pulses with high peak power densities at the optical focus are required to ensure sufficient signal. Contrast in THG microscopy is generated at interfaces where there is a large change in refractive index or third-order non-linear susceptibility^29,30^. Due to higher index of refraction of lipids (R.I.(lipids) = 1.46–1.48 at 1100–480 nm)^31^ with respect to the cytoplasm (R.I. = 1.360–1.390 at 633 nm)^32^, the THG signal is efficiently produced at the interface between the aqueous phase and by lipid-rich structures^33–35^. These include cellular membranes and lipid droplets (LDs).

Lipid droplets are dynamic cellular organelles which play a key role in lipid homeostasis and energy in eukaryotic cells. Studies of lipid droplet physiology in fungi are still in their infancy but their quantitation has relevance to issues in biomedicine, agriculture, industrial waste and the energy crisis. As mentioned above, THG microscopy is a particularly suitable technique for lipid droplet physiology studies^11,35,36^. The advantages of THG microscopy are it is non-invasive, produces inherently confocal images, doesn’t require fixation or external labelling-similar to Raman-based^37–42^, differential interference contrast (DIC)^43^ and light sheet microscopy^37^, and is minimally phototoxic allowing for in vivo studies. A point of difference between Raman-based techniques and THG microscopy is the simpler excitation scheme and minimal risk of aberration artefacts in THG microscopy. Combining THG with fluorescence microscopy is useful to identify the molecular source of the THG-generated signals (i.e. lipophilic fluorescent dyes to target LDs)^4,7,36^. Once THG-associated structures are identified they can be followed using THG microscopy in situ.

Quantitation of images containing LDs can be challenging. The desired parameters include LD number, density, size and morphology. Readily-available image analysis software and programming languages for this purpose are ImageJ, Cell Profiler, Imaris, AMIRA, Volocity, MATLAB, D programming, for both fluorescent images^44–47^ and for lipid droplet images taken by label-free^11,17,20^ techniques. Automated quantitation of lipid droplets uses either thresholding of the images (threshold-based) or watershed methods (morphology-based)^48^, and are usually optimized for a specific cell line. It would be desirable to have a more general image analysis platform that does not require extensive cell-line specific thresholding. In this regard, Image Correlation Spectroscopy (ICS) is a promising method because it is based on measuring spatially-correlated fluctuations. ICS has been applied to confocal images where it measures the spatial variation of fluorescence intensity fluctuations, which can be further related to particle density and aggregation state^49^. On the other hand, ICS has been rarely used for nonlinear techniques, only for two photon excitation fluorescence (TPEF)^50^ or recently for second harmonic generation imaging (SHG)^51^.

The filamentous fungi^52^ are ubiquitous organisms that contribute profoundly to a wide range of ecosystem processes, including decomposition of organic carbon, carbon storage and nutrient transfer. As an invisible and often overlooked part of carbon cycle, filamentous fungi as saprophytes and plant symbionts (mycorrhizal fungi) create a sink for plant organic carbon and distribute it to below-ground hyphal biomass^53^. The oleaginous filamentous fungi have the ability to accumulate large amount of carbon in the form of lipids, more than 20% of their biomass^54,55^ under appropriate conditions. These lipids are considered to be a valuable alternative resource for various biotechnological applications (biodiesel production, high-value chemicals, food/feed additives, and efficient bioremediation of wastewaters)^56,57^, in a bio-based economy. Additionally, the lipid accumulations have been implicated in the resistance of fungi to toxins^58^ and virulence of pathogenic fungi^59^. Moreover, yeast cells modified to lack the lipid droplets entirely, are extremely vulnerable to a variety of stresses^60^ altogether demonstrating that LD studies could potentially lead to novel antifungal treatments. We have chosen for the THG imaging study of LDs the well-known model species *Phycomyces blaekseneanus*, oleaginous fungi from the order Mucorales with very rapid growth (from the spores, through exponential growth phase, to stationary phase in under 36 h). The challenge of utilizing THG imaging for filamentous fungi is that the LDs in filamentous fungi are of rather small dimensions (< 1.5 μm) unlike e.g. in white adipocyte cells where LDs dimensions can reach 100 μm^61^. Our aim is to show that THG microscopy is highly suited for imaging the density and size of LDs in live filamentous fungi. To this end, we will use filamentous fungi in the baseline control condition, with sporadic and small LDs, corresponding to low lipid content conditions^62^, and fungi with denser LDs brought upon by nitrogen starvation-induced autophagy response^63^, the conserved cellular mechanism of molecular recycling^64^. In addition to label-free THG imaging of LDs in fungi, we also present two methods for LDs quantification and analysis. The first method is based on ImageJ/Fiji open source platform, particle analysis tool, which provides measurements of the size, shape and number of LDs. The second method is called Image Correlation Spectroscopy (ICS)^65^, which provides measurements of density and size of particles through spatial autocorrelation analysis.

Our aim is to show that ICS is a good method for quantification of LDs in THG images.

## Materials and methods

### Filamentous fungus strain and growth conditions

A wild-type strain of oleaginous Zygomycetous fungus *Phycomyces blakesleeanus* (Burgeff) [NRRL 1555(-)] was used as the model cell system in this study. For optimal growth of the mycelium, spores at concentration of the order 10^7^ spores/ml were plated on 100 mm Petri dishes at 21–23 °C. Standard liquid minimal (SLM) medium for cultivation contained per liter: 20 g of D (+)-glucose (carbon source), 2 g of L-asparagine·H_2_O (nitrogen source), 5 g KH_2_PO4, 500 mg MgSO_4_·7H_2_O, and microelements/"trace stock" (28 mg CaCl_2_, 1 mg thiamine hydrochloride, 2 mg citric acid·H_2_O, 1.8 mg Fe(NO_3_)_3_·9H_2_O, 1 mg ZnSO_4_·7H_2_O, 300 µg MnSO_4_·H_2_O, 50 µg CuSO_4_·5H_2_O, and 50 µg Na_2_MoO_4_·2H_2_O). The glucose was autoclaved separately, and the final pH of the medium was 4.5. The osmolarity was about 200 mOsm.

**Figure 1:**
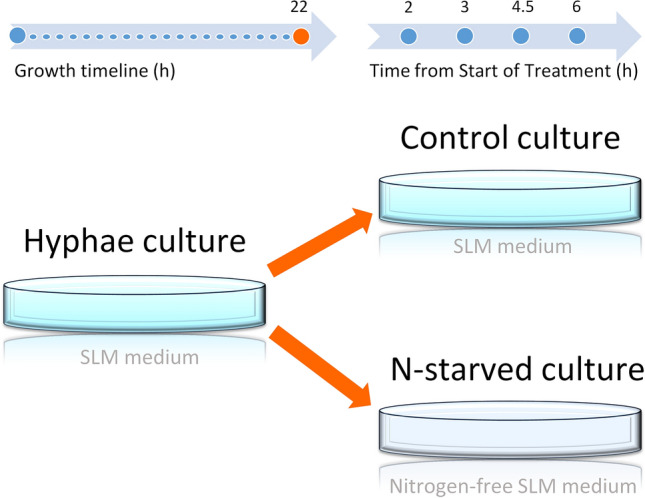
The outline of experimental design of nitrogen starvation. Hyphae cultures are grown in control conditions (Control culture) or in nitrogen-depleted medium (N-starved culture). Time points of sampling are marked as blue dots.

For the nitrogen starvation experiments, the fungi were first grown in the SLM medium and after 22 h were divided into two groups. Group 1 was the control group and group 2 was the nitrogen starved group (N-starved). Fungi from control group 1 were collected by centrifugation (10 min) and resuspended in SLM medium. For group 2, fungal cells were centrifuged (10 min) and resuspended in nitrogen-free medium (SLM medium without L-asparagine) (Fig. 1). The age-matched fungal cultures were imaged at different time points after nitrogen starvation (3, 4.5, 6 h and > 6 h (up to 8.5 h)) at room temperature. All fungal cultures used for imaging were in exponential growth phase (total time from seeding was in the range 24–30.5 h). Data collected from the 6–8.5 h time-points were pooled and represented the group in prolonged nitrogen starvation (labelled 6 h on the graphs).

### Lipid staining

Live fungal cells were stained without chemical fixation. To stain the fungal cells, hyphae in exponential growth phase (26 h) were incubated with 40 ng/mL of Nile Red dye (Acros Organics) for 10 min at 20 °C.

### Nonlinear laser scanning microscopy (NLSM) experimental setup and hyphae imaging

**Figure 2:**
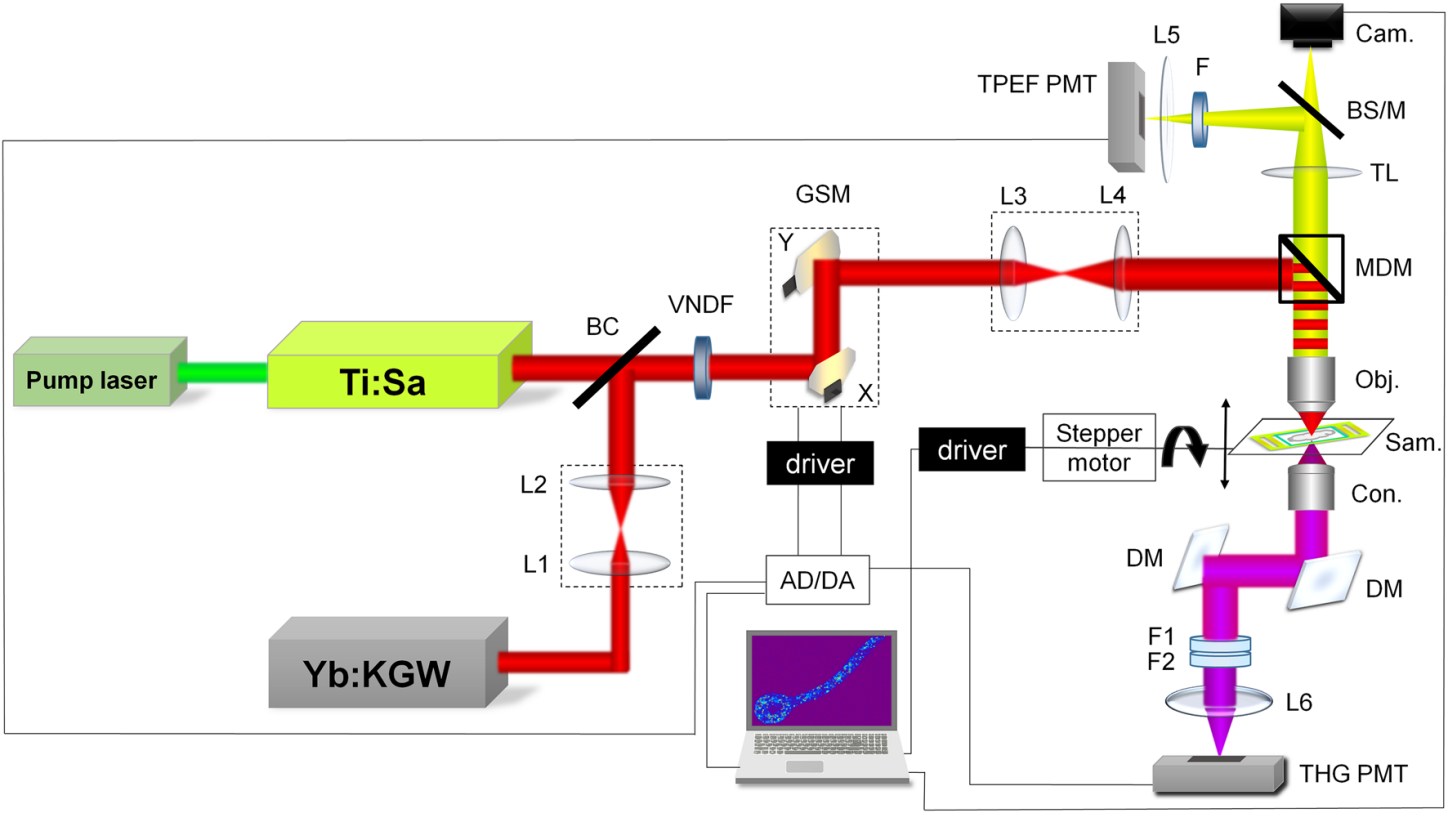
NLSM setup. Ti:Sa—laser for TPEF imaging, Yb:KGW—laser for TPEF and THG imaging, BC—beam combiner, L1 and L2—lenses of 1:1 beam expander for recollimation, VNDF—variable neutral density filter, GSM—galvanometer-scanning mirrors, L3 and L4—lenses of 1:3.75 beam expander for imaging, MDM—main dichroic mirror (cut-off 700 nm), Obj.—microscopic objective 40 × 1.3,  Sam.—sample, Con.—aspheric condenser lens, DM—dichroic mirrors reflective for THG (347 nm) and transmissive for Yb laser (1040 nm), F1—Hoya glass UV filter, peak transmission 340 nm, F2—bandpass filter 275–375 nm, L6—focusing lens, THG PMT—photomultiplier tube for THG signal, TL—tube lens, BS/M—beam splitter or mirror toggle, Cam.—camera, F—VIS filter 400–700 nm for autofluorescence or VIS + 570 nm long pass for Nile Red fluorescence, L5—focusing lens, TPEF PMT—photomultiplier tube for TPEF signal, AD/DA—acquisition card. The scheme was created in Microsoft Power Point 2016 (https://www.microsoft.com/en-us/microsoft-365/powerpoint).

The images of live unstained fungal cells were obtained using a bespoke nonlinear laser-scanning microscope, previously described in references^66,67^, but modified for THG imaging (Fig. 2). For third harmonic generation (THG) imaging of hyphae the following experimental setup, based on significantly modified Zeiss Jenaval upright microscope, was used: Infrared femtosecond pulses were provided by a SESAM mode-locked Yb:KGW laser (Time-Bandwidth Products AG, Time-Bandwidth Yb GLX; Zurich, Switzerland, wavelength 1040 nm, pulse duration 200 fs and repetition rate 83 MHz). The laser wavelength was chosen so that THG signal whose wavelength is 3 times shorter (347 nm) is still in the range of conventional air UV optics. The laser light was first passed through a collimating 1:1 beam expander (L1 and L2) for divergence compensation, and then combined (at BC) with the Ti: Sa laser beam used for TPEF imaging. After that, both beams pass the motorized variable neutral density filter (VNDF) for power regulation and the mechanical shutter. The beams were raster scanned over the sample using two galvanometer mirrors (Cambridge Technologies, 6215H; Bedford, Massachusetts, USA) and a 1:3.75 beam expander (L3 and L4) was used to fill the back aperture of the objective lens and to achieve 4f. configuration. The beams were further directed onto the sample by a short-pass main dichroic mirror (MDM, cut-off at 700 nm) through the high numerical aperture (NA) oil immersion objective lens (Carl Zeiss, EC Plan-Neofluar 40X, NA 1.3). The THG signal was detected in the forward direction (transmission arm), parallel to the direction of laser propagation. First, the signal was collected by high NA aspheric lens (condenser). Then, it was reflected by two dichroic mirrors (DM) that reflect 347 nm but transmit 1040 nm to prevent the laser beam from reaching the detector. Further on, the signal was filtered out from the rest of the laser photons by a bandwidth filter 275–375 nm (Thorlabs FGUV11M) and a Hoya glass UV filter (Newport FSR-U340) with a maximum transmission at 340 nm. The THG signal was detected using a photomultiplier tube (PMT) (Hamamatsu, H7422, Japan), after being focused by a 50 mm focal length lens (L6) onto the entrance window of the PMT.

For the (auto)TPEF imaging a tunable (700–900 nm) Kerr lens mode locked Ti:Sa laser (Mira 900, Coherent Inc. CA, USA) was used, pumped by CW (continuous-wave) frequency doubled Nd:YVO4 laser at 532 nm (VERDI V10, Coherent Inc. CA, USA). The wavelength of the Ti:Sa laser was set to 730 nm for auto TPEF imaging since most of the endogenous fluorophores (NADH, flavins, etc.) can be excited at this wavelength^68^ on the one hand, and because of the technical limitation (laser tunability range and dichroic mirror cut off) on the other hand. The fluorescent signal was collected in back reflection by the objective lens, passed the MDM, tube lens (TL) and filtered out by VIS (400–700 nm) band pass filter (Canon, taken from the camera EOS50D) for the detection of the autofluorescence excited by Ti:Sa laser. Additionally, 570 nm long pass filter (colored glass, unknown vendor) was used for Nile Red fluorescence which is excited by Yb: KGW laser and detected simultaneously by THG signal. TPEF signals were detected after being focused by 50 mm focal length lens (L5) onto the entrance window of the TPEF PMT.

The acquisition was performed by National Instrument card USB-6351 at the rate of 1.2 M sample/s. This enabled high enough frame rate at low resolution for live monitoring, for instance 3 frames per second at 256 × 256 pixels with 6 averages. For high resolution images, it takes 30 s for 1024 × 1024 image with 30 averages. The lateral and axial resolution of the microscope with 40 × 1.3 objective lens were estimated to be 300 nm and 1000 nm, respectively.

Bright field images were taken with a Canon EOS 50D digital camera (Tokyo, Japan) whose CMOS sensor was placed at the image plane of the tube lens. Toggle switch BS/M enables utilization either of camera for bright field or TPEF PMT for fluorescence imaging.

A specially designed sample holder was used, that enables hyphae with the growing medium to be placed between two coverslips in order meet the criteria for the best NA of the objective lens, but also to avoid losses of the UV THG signal by thick deck glass (Supplementary Figs. S1 and S2 show different imaging conditions of hyphae that were tested in order to find the best one). The #1.5 coverslips (170 μm thickness) were used. 20 μl of hyphae suspension was used to keep the hyphae alive. The holder was placed between objective lens and the aspheric condenser on the motorized table that can be translated in steps of 0.3 μm along the beam propagation direction (z axis) for optical slicing of the sample and 3D imaging.

In control versus N-starved group imaging, time points were gathered sequentially. Using a label-free imaging technique, such as THG, enabled us to take images of samples with minimal delay after taking fungi from the culture. The overall time a sample culture was kept under the microscope to acquire at least 3 THG images of live hypha was between 25 and 37 min. Effectively, time points for control and treatment were offset for 30–40 min on one experimental day and on the next day, offset in opposite direction to the other. The exact ranges of time of growth (mean and standard deviation) for all hypha included in experimental groups are collected in Supplemental Fig. S4.

### Image analysis

THG image analysis of lipid droplets in 2D was performed using ImageJ (W. Rasband, National Institute of Health, Maryland, USA, http://imagej.nih.gov/ij/). Algorithms written in MATLAB (in-house-created code) and VolView software were used for 3D and 4D image processing. Two methods for image analysis were used to quantify LDs number and size, Particle Size Analysis (PSA) and Image Correlation Spectroscopy (ICS). Details of both procedures are in the Supplementary Information.

### Statistics

For quantitative image analysis, images of individual hypha under control conditions (n = 44) and after nitrogen starvation (n = 17) were obtained from 6 independently grown cultures. GraphPad Prism was used for graphing and statistical comparisons. The boxes of the box and whisker plots are enclosed by the 25th and 75th percentile range with the line representing the median; the whiskers are extending to the minimal and maximal value, respectively. Histograms of number of LDs were generated from all LD diameters in each group with 0.3 µm binning and each bin value was divided with the sum of hypha areas in the group. Errors in histograms of Number of LDs/hypha area were calculated as: Relative Error (binned N/area) = Relative Error (Number of LDs/hypha area) + Relative Error (Area), and Relative Error (binned N/area) was multiplied by value of Number of LDs/hypha area for that bin. Two-way ANOVA with multiple comparisons and Holm-Sidac correction and unpaired two tailed t test with Welch's correction for unequal variances, were used for the calculation of statistical significances. Where appropriate, unpaired two-sided Mann–Whitney test with was used instead. Confidence level for statistical significance was: 0.05 (*), 0.01 (**), 0.005 (***), 0.0001 (****).

## Results

**Figure 3:**
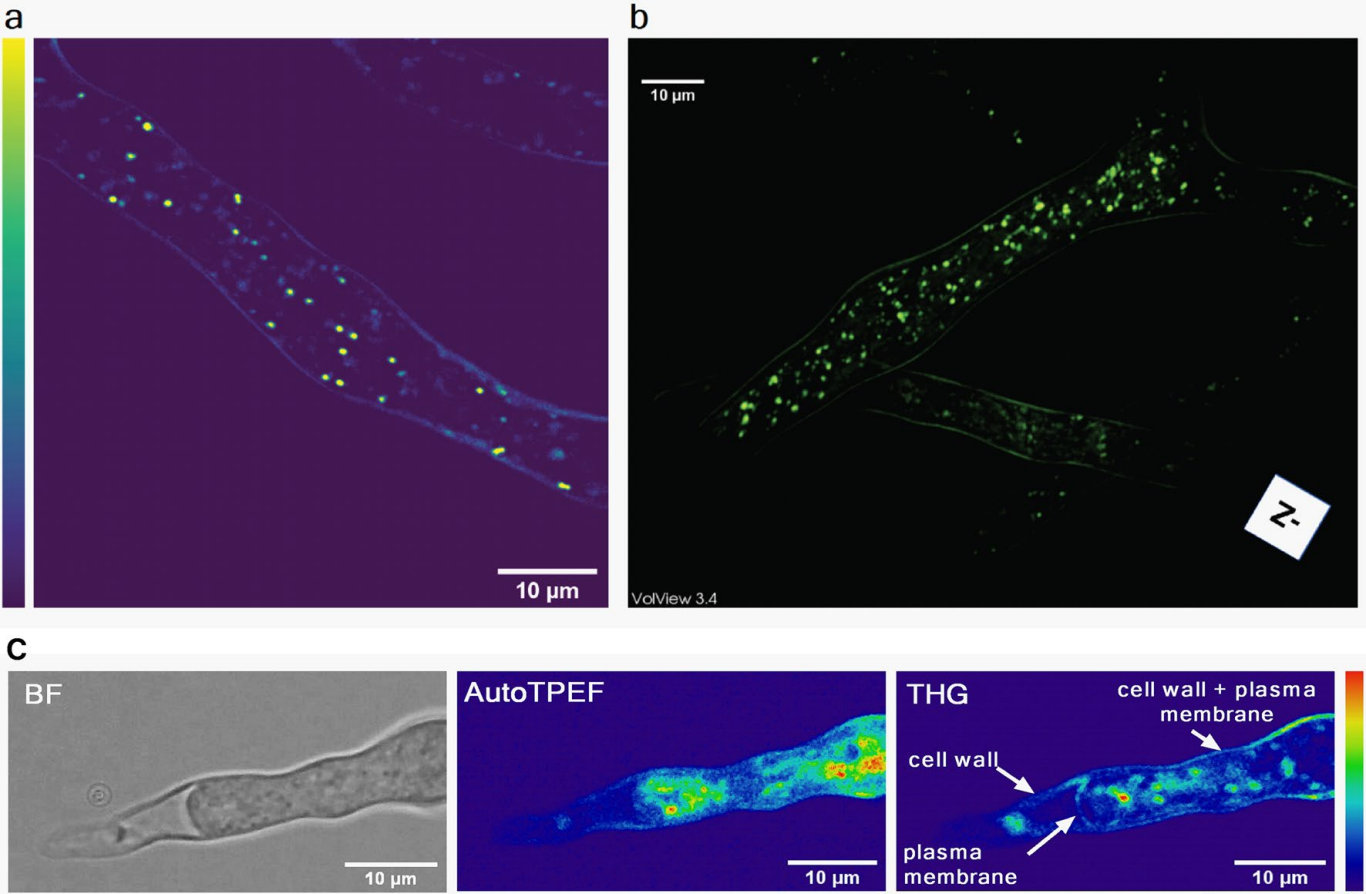
Label-free imaging of *Phycomyces blakesleeanus* hyphae from the exponential phase in SLM. (**a**) one THG slice; (**b**) 3D model built out of 23 THG slices 0.9 µm apart. The average laser power at sample plane was 23–26 mW. (**c**) Multimodal imaging: bright field (BF) (left), autoTPEF (middle) and THG (right) images of the same live unlabeled hypha. The hypha was plasmolyzed and the retracted plasma membrane is solely visible in the THG image. The average laser power at sample plane was 2.7 mW (TPEF) and 55 mW (THG). Color intensity bar for both, TPEF and THG signals: deep blue—the lowest signal, red the highest signal. All the images were taken with Zeiss 40 × 1.3 oil objective lens.

THG images, one slice (2D) and 3D reconstruction, of unstained live *P. blakesleeanus* hyphae in exponential growth phase are shown in Fig. 3a,b, respectively. The THG signal at the cell circumference originates from chitinous cell wall and plasma membrane which follow the cell wall shape. In the cytoplasm, various entities that produce THG signal are visible. The hyphae were placed in the liquid growth medium between two coverslips. The high resolution of the microscopic system (diffraction limited), the thickness of the hyphae (ca 10 µm) and transparency of the medium make possible the whole hyphae to be optically sectioned and a 3D model to be reconstructed (Fig. 3b and Supplementary Video S1 in the Supplementary Information). It is obvious that strong THG signal features are prominent among all the entities in the cytoplasm. According to the literature these are most likely lipid droplets since they have a large index of refraction in comparison with the rest of cytoplasm. In addition, the power dependence of the THG signal originating from LDs is provided in Supplementary Material (Fig. S3).

The cell wall and plasma membrane are separated by a very small distance which is not resolvable in the images of native hyphae obtained by diffraction limited techniques (resolution of approximately 250 nm). To visualize the cell wall and the plasma membrane separately, we plasmolyzed the hyphae so the plasma membrane was retracted from the cell wall at a resolvable distance (Fig. 3c). The retracted cytoplasm is clearly visible in bright-field (Fig. 3c left) and autoTPEF images (Fig. 3c middle), but the plasma membrane can be solely distinguished only in the THG image (Fig. 3c right) since its refractive index is different from the cytoplasm.

### There is no significant overlap of AutoTPEF and THG signal in the hyphae

**Figure 4:**
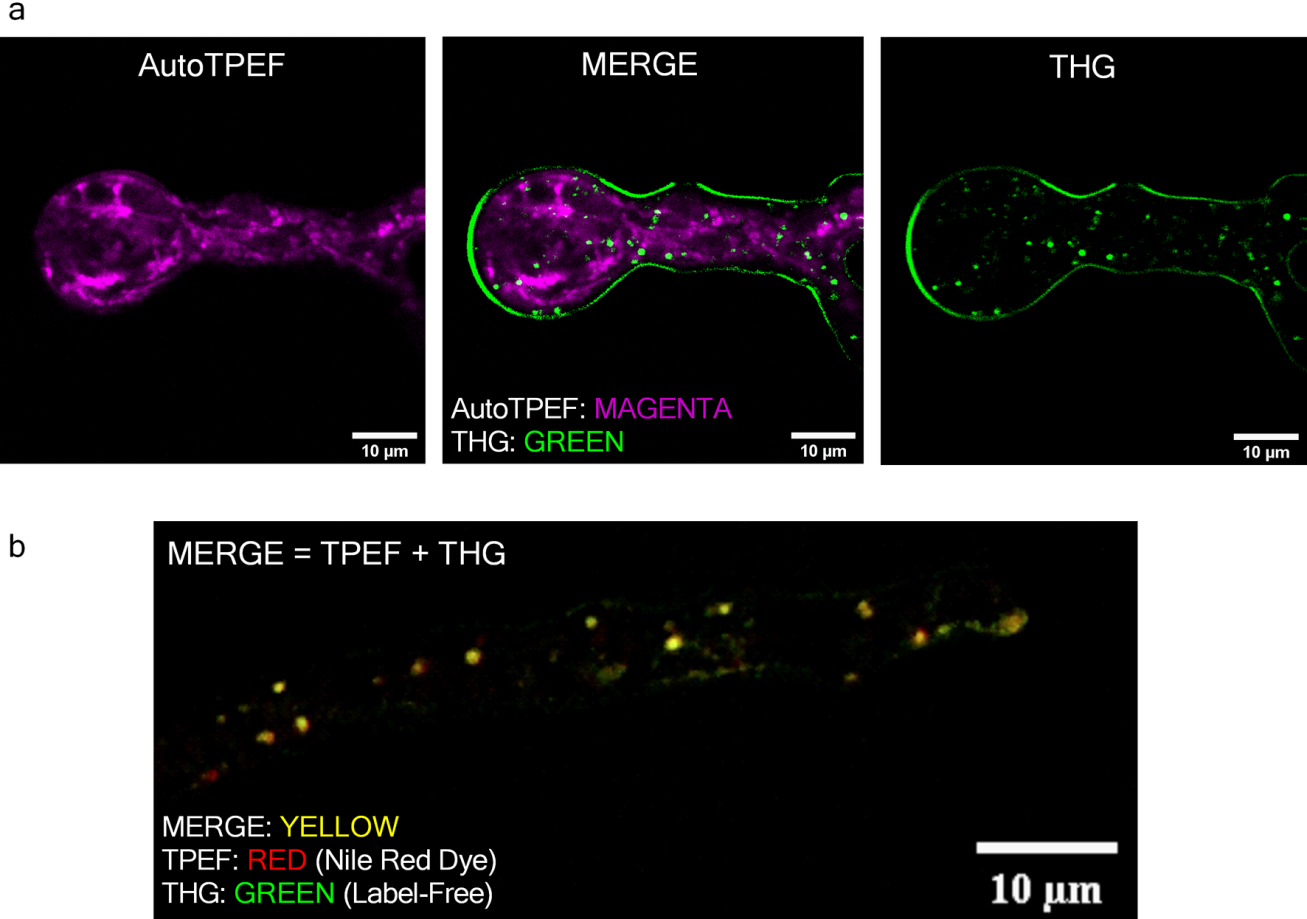
TPEF and THG images of *Phycomyces blakesleeanus* exponential growth phase hyphae in standard liquid medium show that the predominant source of spot wise THG signal are lipid droplets. (**a**) Merged autoTPEF and THG images of same unlabeled live hypha showing that there is no overlap of autoTPEF and THG signal. The average laser power at sample plane was 28 mW at 1040 nm (for THG) and 3.4 mW at 730 nm (autoTPEF). (**b**) In vivo colocalization of stained LDs imaged by TPEF and LDs imaged by THG modality. Average laser power at sample plane was 32 mW for both THG and TPEF at 1040 nm. Pearson’s correlation coefficient R_total_ = 0.844 (ImageJ, The Colocalization Threshold plugin). All images were taken with Zeiss 40 × 1.3 oil objective lens.

While THG imaging is not necessarily specific for LDs, because the THG signal is produced by any refractive index change, LDs still produce significantly higher THG signal in comparison with other structures in the cytoplasm of a cell like *P. blakesleeanus*. This fact can be used to extract LDs in a cell, over a broad but still much lower signal range than other cytoplasm entities. As the very first step toward the confirmation that high THG signal features in unlabeled live *P. blakesleeanus* are LDs, we performed the imaging of the same hyphae by detecting auto fluorescence signal upon two photon excitation at 730 nm (Fig. 4a left). In order to ensure that high THG signal entities (Fig. 4a right) are not artifacts that might be caused by e.g. high laser intensity damage, we merged the two images, THG and autoTPEF (Fig. 4a middle) showing clearly there is no significant increase of TPEF signal at the same locations. The hyphae were in exponential growth phase, as in Fig. 3.

### Colocalization of lipid droplets signal imaged by TPEF and THG

Whilst many label-free imaging studies on various biological samples have shown that strong THG contrast in the cytoplasm arises mostly from LDs^11,35,36^, in the case of *Phycomyces blaekseneanus* THG imaging has never been applied to this type of organism.

To prove firmly that the cytoplasmic puncta in THG images of hyphae are LDs we performed colocalization experiments (Fig. 4b). The hyphae were stained by Nile Red dye which is considered as a standard for lipids^69^. The TPEF of Nile Red dye was excited by the same laser used for THG and the TPEF signal was collected through 400–700 nm band pass and 570 nm long pass filters, which effectively isolates the fluorescence signal to the 570–700 nm spectral region. The laser beam was focused with the Zeiss Plan Neofluar 40 × 1.3 objective lens, and both, TPEF and THG signals were detected simultaneously. Before the measurement, a very small volume of the sample (10 μl of fungi suspension) was added between two coverslips. This enables hyphae to stay alive during the imaging but also to be immobilized as close as possible to the coverslip thus achieving the best possible resolution.

The quantitative comparison of the TPEF and THG images (colocalization analysis) was performed based on Pearson’s correlation coefficient and Image Cross-Correlation Spectroscopy (ICCS). Pearson’s correlation coefficient was in the range 0.74 < R_total_ < 0.88 (ImageJ, The Colocalization Threshold plugin). According to the ICCS analysis, the fraction of THG-detected clusters interacting with the TPEF-detected clusters was 0.89 indicating a high degree of spatial correlation between fluctuations generated from the lipid probe and THG signal. The degree of colocalization obtained in our work is in accordance or higher with those obtained in label-free imaging on live and in some fixed samples^7,70^.

Based on the colocalization experiment (Fig. 4b) and the results shown in Fig. 4a one might consider that most round bright features in THG images of *Phycomyces blaekseneanus* are the lipid droplets.

### THG image analysis and quantification of lipid droplets

**Figure 5:**
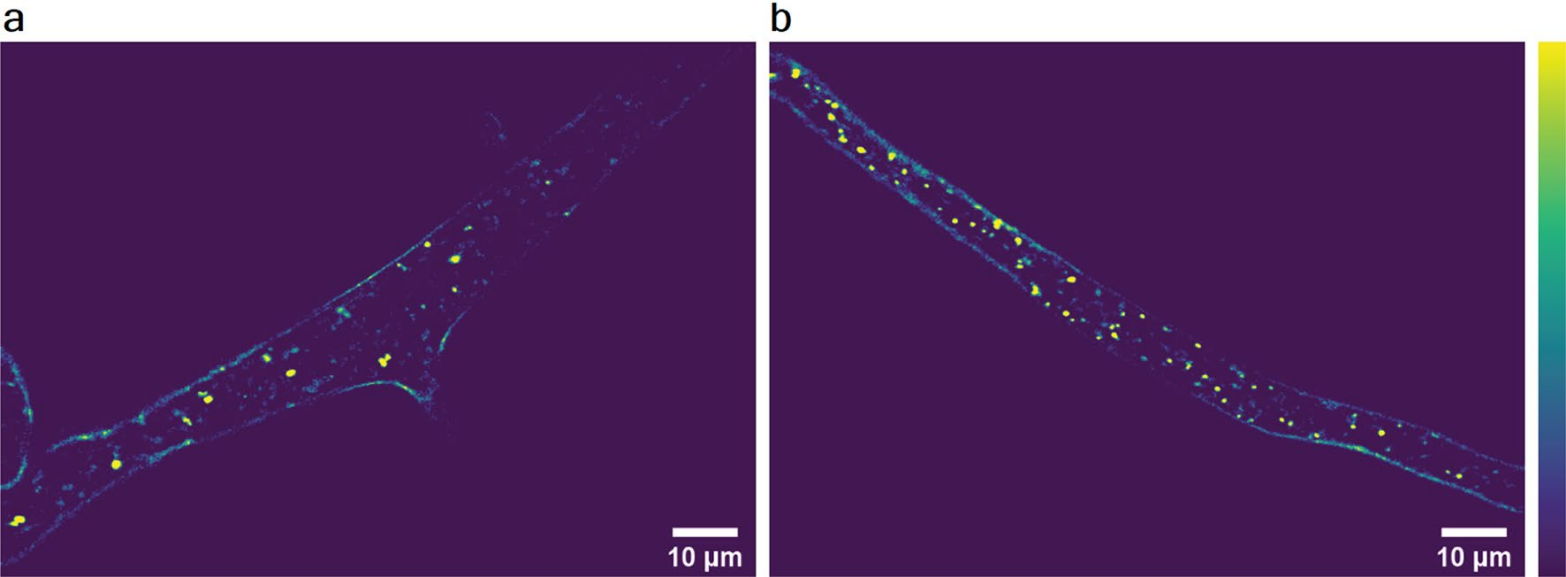
THG images of N-starved hyphae. (**a**) control hyphae; (**b**) N-starved (4.5 h duration of growth in nitrogen-depleted conditions). Both images were taken with Zeiss 40 × 1.3 objective lens whilst average laser power at sample plane was 24 mW (in A) and 20 mW (in B). Violet-lowest THG signal, yellow—highest THG signal.

For the quantification of LDs, we analyzed a small set of THG images by Particle Size Analysis and Image Correlation Spectroscopy (both available in ImageJ). To test and compare the two methods we used hyphae cultures grown in completely nitrogen-depleted media (N–starved) and their age-matched sister cultures from the same batch grown in standard media as a control. Nitrogen limitation is known to cause autophagy in filamentous fungi^71^, leading to alterations in lipid metabolism and an increase in the number of LDs^72,73^. We performed THG imaging on hyphae in exponential growth phase, alternating between control (Fig. 5a) and N-starved (Fig. 5b) age matched hypha batches. From Fig. 5 it is obvious, even by the bare eye, that there is significant increase in LD number after nitrogen starvation for 4.5 h. Once we confirmed that we obtained the expected increase of LD number, we went ahead to test the two methods of quantification and the sensitivity of THG imaging for LDs detection.

**Figure 6:**
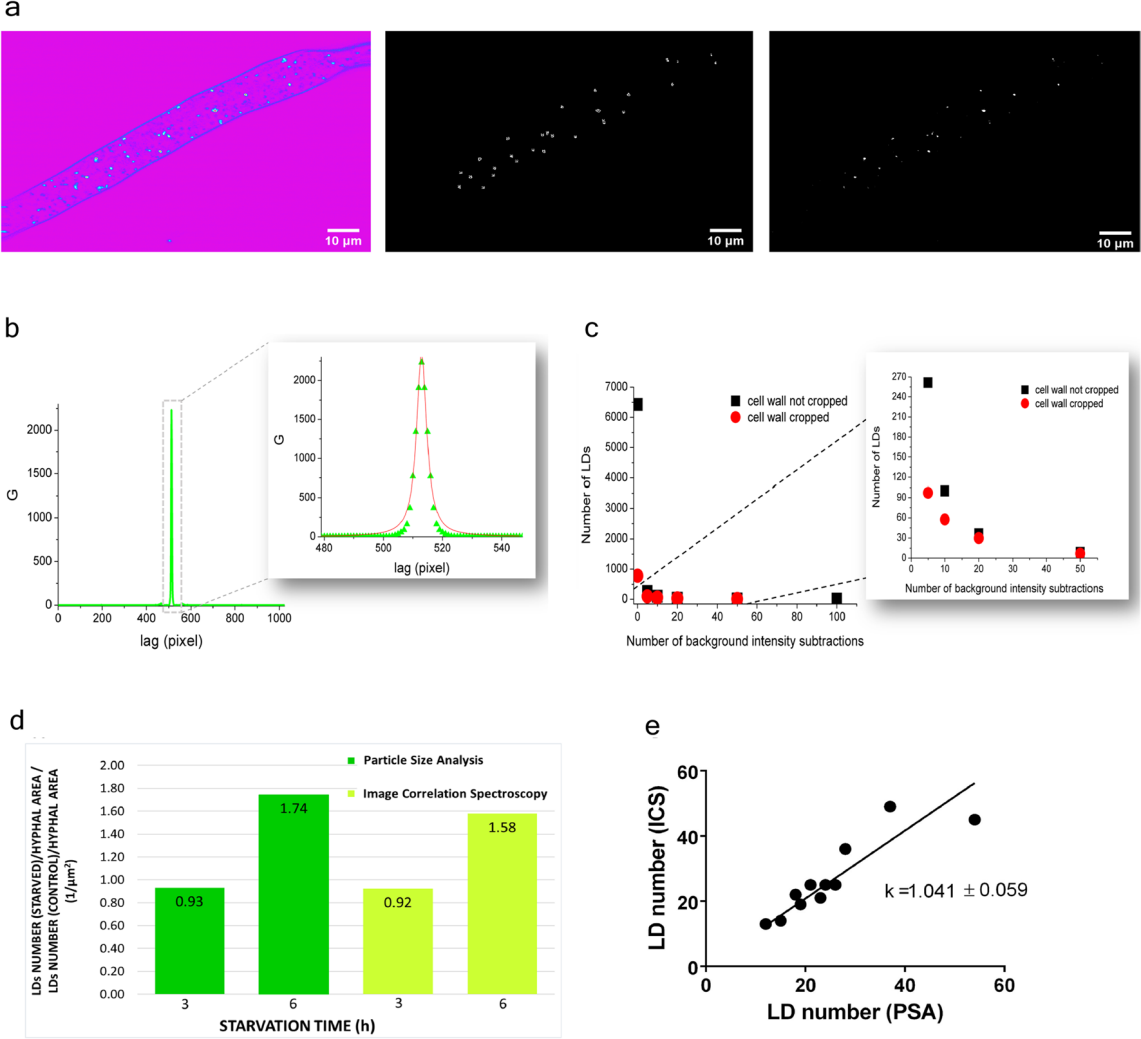
Image Correlation Spectroscopy (ICS) and Particle Size Analysis (PSA) on THG images. (**a**) Processing for PSA and ICS analysis of the same THG image. Left: The unprocessed THG image of *Phycomyces blakesleeanus* exponential growth phase hyphae in standard liquid medium; middle: 8-bit mask obtained in Particle size analysis; right: background subtracted image for ICS analysis. The image from left (unprocessed THG image) was processed by applying 20 × background subtractions. Both images are displayed at full dynamic range (8 bits). THG image was taken with Zeiss 40 × 1.3 objective lens, while average laser power at sample plane was 27 mW. (**b**) ICS analysis: The autocorrelation function (G curve) taken as the plot through the center of intensity correlated THG image of a live and unlabeled hyphae. The autocorrelation curve was fit to a Lorentzian function to extract FWHM value as described in Methods section. (**c**) ICS analysis, the effect of the cell wall removal: The number of LDs obtained from the G curves after each background subtraction for the THG image where the cell wall was manually cropped (red circles) and for the same THG image where cell wall was not cropped prior to the background subtractions (black squares). (**d**) Comparison of ICS- and PSA—derived data obtained from the same set of THG images of cultures N-starved for 3 h and 6 h and their age-matched controls (n = 3 for each group). The ratio of the number of LDs per unit hyphal area, in N-starved hypha to the number of LDs per unit hypha area in age- matched controls. (**e**) The agreement of LD number quantification obtained by ICS and PSA. For each image, ICS-obtained LD number is plotted against PSA-obtained LD number for that image. Data for both graphs were obtained from label-free THG images, whose analysis is presented in Table 1.

For the PSA method (available in ImageJ as “Analyze particles”) the raw THG image (Fig. 6a left) was thresholded and converted to an 8-bit mask, upon which the program automatically counted the number of “particles” representing LDs in images analyzed (Fig. 6a middle). In addition to the number of particles, the diameter and area were quantified as well.

Because of the thresholding and limited resolution of the image (pixel size), the PSA might be insensitive to very small or weak signal entities. As the result, some emerging LDs might be omitted and not shown in the final results. To resolve this issue, we performed ICS which extracts the information on particle properties (number and size) based on the spatial fluctuations of the signal intensity in the images. ICS is also applicable to images that are diffuse.

Due to the morphology of the hyphae, it was necessary to pre-process THG images before applying the ICS analysis. Cell wall of hyphae was removed from the image since it hinders the correlation analysis (producing the pedestal at the G curve) because of the sharp discontinuity in intensity at the periphery of the hyphae along the whole circumference. We applied multiple subtractions of the background (average pixel intensity of ROI outside the hypha) until the wall disappears^74^. The latter procedure is depicted in Fig. 6a right and it is obvious that the THG signal from the majority of LDs is much more intense than the signal from the wall (approx. > 10x).

After removal of the cell wall, image correlation procedure was performed in Image J. As the result one obtains a spatial autocorrelation image from which the G curve is extracted by taking an intensity profile through the center of the image. An example of a G curve is shown in Fig. 6b. The number of LDs was calculated using the following formula:

$$N_{LD} = \frac{{N_{pix} \cdot N_{pix} }}{{r^{2} \pi \cdot G\left( 0 \right)}}$$

where N_pix_ is the pixel size of the 2^n^ × 2^n^ image (where n is an integer), r is mean radius of LDs taken as half of the FWHM of the G curve, and G (0) is maximal value of the G curve. r and G (0) are extracted from the Lorentzian fit of the G curve (Fig. 6b insert). It should be noted that the morphology of the LDs differs substantially from the morphology of the clusters which are usually examined by ICS analysis. Thus, in our case, multiple subtractions of the background do not lead to the flattening of the curve G versus number of subtractions as might be expected^74^. The flattening of the G curve shown in the reference 74 is used as criterion how many times the background has to be subtracted before ICS is applied. Our criteria for the number of background subtraction were: (a) cessation of a significant reduction in the number of LDs after each subsequent subtraction (Fig. 6c, black squares), (b) approximate matching of the number of LDs per hyphae with PSA and (c) experience (the cell wall disappears from the image observed by the eye). Upon examination of tens of images, both control and treated hyphae, we concluded that, on average (depending on initial image quality), 20 consecutive subtractions were sufficient for reliable ICS analysis.

To check whether the extra removal of the cell wall would give different number of LDs, we performed manual removal of the cell wall solely. It was done by delineation and cropping prior to the multiple background subtractions. After 20 consecutive background subtractions, this method does not give substantially different results in the number of LDs compared to images where the cell wall was not manually cropped (illustrated by the graph in Fig. 6c).

### LDs analysis by PSA and ICS

A comparison of LD number and size obtained by ICS and PSA is in Table 1. The number of LDs per area of hyphae is approximately the same on average, but mean diameter obtained by ICS is slightly lower. This discrepancy might be explained because of different definitions for the object size used in those two methods.

**Table 1:** A comparison of the number and size of lipid droplets obtained by the quantification analysis of the two methods, ICS and PSA.

Control/Treatment	Image correlation spectroscopy		Particles size analysis	
	Number of LDs/hyphae area (1/μm^2^) × 10^−3^ (mean ± SE)	Mean LDs diameter (μm) (mean ± SE)	Number of LDs/hyphae area (1/μm^2^) × 10^−3^ (mean ± SE)	Mean LDs diameter (μm) (mean ± SE)
Control cells for 3 h N-starvation	16 ± 1	0.56 ± 0.06	16 ± 2	0.74 ± 0.02
3 h N-starved cells	15 ± 5	0.60 ± 0.10	15 ± 6	0.74 ± 0.03
Control cells for 6 h N-starvation	15 ± 4	0.52 ± 0.08	13 ± 3	0.78 ± 0.02
6 h N-starved cells	24 ± 3	0.46 ± 0.02	22 ± 4	0.74 ± 0.04

n = 3 for each group presented.

To estimate the change of LD number in treated hyphae, we calculated the ratio of LD number per area in treated hyphae in respect to control ones (Fig. 6d). The total number of LDs after 3 h of starvation shows no significant change. With longer starvation time the number of LDs increases by more than 50%.

Using ICS analysis, number of features counted was 80 ± 12% of the LD number that was found by visual inspection (n = 12) and in close correlation with the data obtained by PSA. When numbers of obtained LDs by both methods are plotted for each individual image as a separate point (Fig. 6e), the regression line has a slope close to 1, confirming that ICS is equally reliable as PSA method in detecting and counting LDs. The coefficient of regression R^2^ was approximately 0.8.

### Nitrogen starvation induced changes in lipid droplet number and size, as quantified from THG images

To fully use THG imaging (exposure time for an image takes maximum 30 s for 1024 × 1024 pixels image with 30 averages) and subsequent analysis as a LD assay, we performed a set of imaging and measurements across time, from filamentous fungi cultures, grown in nitrogen-depleted and control conditions. Fungi cultures were imaged after growing at least 2 h post start of the treatment (nitrogen starvation or control), precautionary step to avoid possible effects of manipulation during preparation for the start of treatment (e.g. centrifugation).

**Figure 7:**
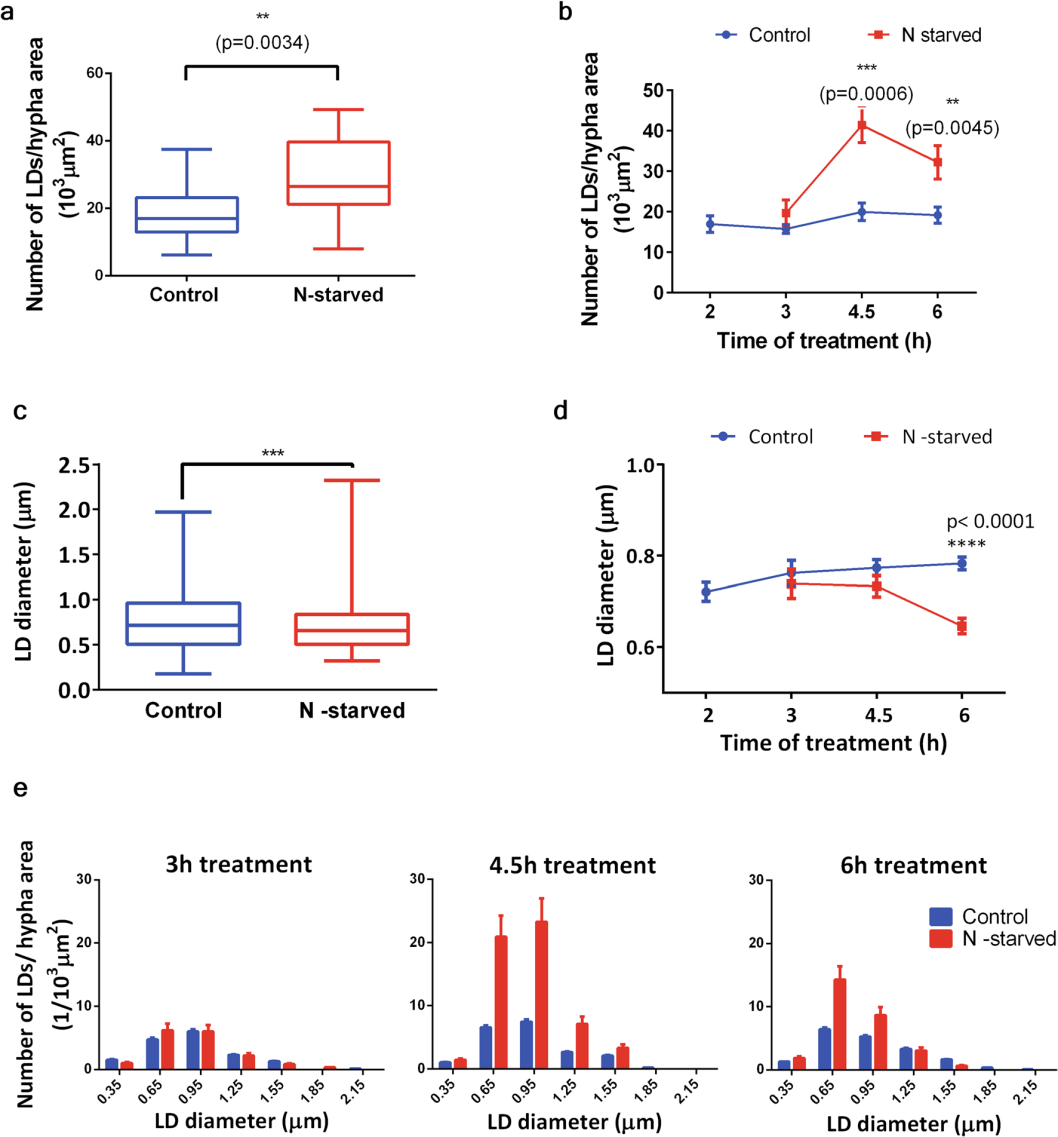
Quantification of LDs from THG images of *Phycomyces blaekseneanus* hypha. Hypha were cultured without nitrogen or in standard liquid media for 2–6 h (or longer up to 8 h) after the start of treatment. Obtained THG images of LDs were analyzed by PA. n = 6 independent cultures. (**a**) N-starvation increases number of LDs per unit area. LD number obtained from the individual hypha is normalized to hypha area (in 10^3^ µm^2^). Control (n = 44), N-starved group (n = 17). The box and whisker plots, enclosed by the 25th and 75th percentile range, median line with whiskers extending minimal to maximal value. Unpaired t test with Welch's correction, two tail, *p* = 0.0038. (**b**) Time course of LD number/unit area, showing that the increase of LD number by N-starvation is significant at 4.5 h (*p* = *p* = 0.0006) and later times (*p* = 0.0045), compared to corresponding control. Two-way ANOVA, with Holm-Sidac correction. Mean ± SE, n_(Control)_ = 8; 7; 11; 21 for time points (in h), respectively: 2; 3; 4.5; 6. n_(N-starved)_ = 6; 3; 7 for time points (in h), respectively: 3; 4.5; 6. (**c**) N-starvation decreases diameter of LDs. LD diameters from Control (n = 1205) and N-starved group (n = 431). The box and whisker plots, enclosed by the 25th and 75th percentile range, median line with whiskers extending minimal to maximal value. Mann–Whitney (*p* = 0.0008), two-tailed. (**d**) Time course of LD diameter changes, showing that the decrease by N-starvation is significant only at long starvation times. Two-way ANOVA, Holm-Sidac correction (*p* < 0.0001), compared to corresponding control. Mean ± SE, n_(Control)_ = 176; 124; 302; 571 for time points (in h), respectively: 2; 3; 4.5; 6. n_(N-starved)_ = 100; 118; 214 for time points (in h), respectively:3; 4.5; 6. (**e**) Differential distribution of increased number of LDs after 4.5 h and 6 h N-starvation. The largest LDs are lost at longest starvation times. Histograms of LD diameter distributions, 0.3 µm binning, for Control and N-starved group. Number of LDs in each bin of the histogram is divided by sum of hypha area of the appropriate group. Errors are calculated as stated in Methods section. Numbers on x axes represent the upper bin limit.

The number of LDs per unit cell area (Number of LDs/hypha area) in all imaged hypha in fungi cultures grown in nitrogen depleted media (N-starved) was significantly larger compared to the entire group of control culture hypha (Control) (Fig. 7a). To elucidate the time course of observed induction of increase in LD number, the Control and N-starved groups are broken down to duration-from-start-of-treatment groups each, and values of Number of LDs/hypha area plotted across time (Fig. 7b). The Number of LDs/hypha area in Controls remained almost the same during the time of observation, with the slight, not significant, trend of increase towards the later growth time points (Fig. 7b). N-starved had similar Number of LDs/hypha area to corresponding Control only at the 3 h of treatment time point. We detected twofold increase of Number of LDs/hypha area after 4.5 h treatment, compared to corresponding Control. Significant increase of Number of LDs/hypha area in N-starved hypha, compared to corresponding Control hypha, persisted at longer times of treatment (Fig. 7b).

The average diameter of LDs was significantly reduced in N-starved cultures, compared to controls, when entire groups were compared regardless of treatment time (Fig. 7c). As it can be seen from the time course graph (Fig. 7d), average LD diameters were approximately the same from the 2 h treatment time to the longest treatment time in Controls. They were also same in 3 h and 4.5 h N-starved hypha and their corresponding Controls. The effect of N-starvation on average LD size becomes clear only after 6 h or more of treatment (Fig. 7d).

The histograms of LD diameters, graphed as Number of LDs/hypha area (Fig. 7e) for the 4.5 h and 6 h time-of-treatment groups, reveal that LDs smaller than 1.6 µm are more numerous in N-starved groups than in corresponding Controls for 4.5 h time point, while at 6 h, only the number of LDs smaller than 1 µm is increased. LD average diameter change between 4.5 and 6 h N-starvation groups seems to be a result of significant loss of population of LDs larger than 0.6 µm during prolonged growth in N-starving conditions. To summarize, the overall change in LDs during growth without available nitrogen is found to be an increase in number of LDs between 3 and 4.5 h time point, followed with the loss of population of larger-than-average LDs during prolonged starvation.

## Discussion

Once considered to be passive lipid storage agglomerations, lipid droplets are now recognized as dynamic cellular organelles, serving as ubiquitous central hubs of energy and lipid homeostasis in eukaryotic cells^75^. Studies of lipid droplet physiology in fungi, although still scarce^76^, harbor promise of providing novel solutions for a number of important issues: mitigation and modulation of fungal resistance to fungicides and stress, securing the food safety, better understanding how to use fungi as a crucial component of sustainable organic waste reuse and conversion to energy source, to name a few. *Phycomyces blakesleeanus*, model fungus used in our study, belongs to *Mucormycota,* the phylogenetic group of fungi able of forming arbuscular mycorrhiza and other mutually beneficial symbiosis^77^ with terrestrial plants^78^. During fungi-plant mutually beneficial interaction, a fungi transports nitrogen to a plant, and receives up to 30% of organic C compounds synthesized by a plant^78^ in return. It is known that organic molecules sent from plant to fungi are lipids^79,80^, and that lipid droplets form in large amounts in hypha adjacent to the area of contact with the plant^81^. Similar to *Phycomyces*, arbuscular mycorrhizal fungi can accumulate significant amount of acquired organic carbon in the form of lipid droplets^82^. THG imaging of LDs as described here is a method that could be directly applied to living mycorrhizal fungi related to *Phycomyces*, without the need for any modification of the protocol, or other staining.

The fungi culturing conditions used in our study resulted in a fairly modest accumulation of lipid droplets, as expected^62^. THG imaging analysis enabled us to watch and quantify changes in lipid droplet number, brought upon by complete removal of nitrogen, from such low density/diameter baseline. As expected, complete omission of nitrogen induced only a transient increase in number of lipid droplets, followed by lipid turnover^83^. THG imaging analysis detected the significant decline of lipid reserve at late stages of growth. Altogether, this shows the usefulness of THG imaging approach for broader exploration of LD in filamentous fungi under various living conditions.

Optical imaging techniques are commonly used to study lipid droplets in vivo, but lipids usually have to be labeled with various dyes. On the other hand, prolonged imaging using fluorescent dyes can be phototoxic to cells and may perturb metabolic processes, including lipid metabolism. Hence, label-free imaging methods, are advantageous for the study of living cells^84–86^.

THG imaging, a label-free method we have applied to live hyphae of oleaginous fungi *Phycomices blakesleeanus*, generated images with the characteristic spots of high THG signal intensity attributed to lipid droplets as the products of normal and stressed cellular physiology. Several lines of evidence support this attribution. First, the steep change of refractive index between lipids at the interface of lipid droplets and the rest of the cytoplasm generates high intensity THG signal, according to literature^11^. Second, to exclude possible laser-damaged spots that would produce high THG signal, we performed TPEF imaging of unstained hyphae showing that autoTPEF images are devoid of any prominent spots, present on a THG image of the same hypha. Third, we have performed colocalization experiments where the hyphae were stained with lipid specific dye and imaged by both, TPEF and THG method. The spots at the both images were mostly overlapped which verifies that the spots contained lipids. In addition, following the same logic of steep changes of refractive index, we have shown that the cell wall and the cell membrane in label-free hyphae can be imaged and distinguished by THG method.

There are a number of caveats to be discussed regarding the imaging of lipid stains. Because of the simultaneous detection of both (TPEF and THG) signals restricted number of dyes for live imaging could be used. In this study fixation was not a choice since it alters the structure of LDs^87^. The dye used in this study, Nile Red, might be not so specific for LDs and it can bind to other bodies and structures in the cell^88^. The signal originating from other structures than LDs can bleed into the detection band which eventually might affect the colocalization degree. In addition, the degree of colocalization is further deteriorated by the strong THG signal from the cell wall. The THG imaging requires significantly higher laser powers in comparison to the TPEF imaging. Because of that, one has to make a trade-off in terms of applied laser power when detecting both signals simultaneously. The price paid for this trade-off is the loss of some structures (e.g. small LDs, otherwise visible at higher laser powers) in THG images and appearance of weak, blurry TPEF signal from out of focus LDs (otherwise not visible at lower laser powers).

To extract quantitative data from THG images, two methods for image analysis were applied, Particle Size Analysis (PSA) and Image Correlation Spectroscopy (ICS). Both methods can quantify the number of lipid droplets and their average size (diameter). Since ICS was primarily developed for fluorescent images and cluster analysis and to the best of our knowledge it was not used so far for THG images, we have tested it by comparing the results to the PSA. The test was performed on the images of the hyphae under normal and stressed (nitrogen starvation) circumstances. The nitrogen starvation is known to cause increased number of lipid droplets^72,73^ which was confirmed by both methods and the agreement between numbers obtained by both methods was good.

Overall, the proposed imaging method (THG) and the method of image analysis (ICS) was shown to be suitable for label-free in vivo studies of lipid droplets of oleaginous fungi. Application of THG method to future studies of lipid droplet dynamics in fungi could help to advance basic understanding of fungi cellular physiology, and then, of processes involved in the cycling of carbon in nature.

## Supplementary Information

Supplementary Information 1.

Supplementary Video S1.

## Data Availability

The data available upon a reasonable request to the corresponding author.
