# Supplementary material for: Label-free third harmonic generation imaging and quantification of lipid droplets in live filamentous fungi
Source: Sci Rep. 2022 Nov 5;12:18760. doi: 10.1038/s41598-022-23502-4 (PMC9637149; doi:10.1038/s41598-022-23502-4)
Supplement: Supplementary file 1 — Supplementary Information 1. [file 41598_2022_23502_MOESM1_ESM.pdf]

SUPPLEMENTARY INFORMATION:

Label-Free Third Harmonic Generation Imaging and Quantification of Lipid Droplets in Live Filamentous Fungi

Tanja Pajić[1], Nataša V. Todorović[2], Miroslav Živić[1], Stanko N. Nikolić[3], Mihailo D. Rabasović[3], Andrew H. A. Clayton[4], and Aleksandar J. Krmpot[3]*

[1]Institute of Physiology and Biochemistry, Faculty of Biology, University of Belgrade, Studentski trg 16, 11158 Belgrade, Serbia

[2]Institute for Biological Research "Sinisa Stankovic", University of Belgrade, National Institute of the Republic of Serbia, Bulevar Despota Stefana 142, 11000 Belgrade, Serbia

[3]Institute of Physics Belgrade, University of Belgrade, Pregrevica 118, 11080 Belgrade, Serbia.

[4]Optical Sciences Centre, Department of Physics and Astronomy, School of Science, Computing and Engineering Technologies, Swinburne University of Technology, Melbourne, Victoria 3122, Australia

*Corresponding author: Aleksandar J. Krmpot, Institute of Physics Belgrade, University of Belgrade, Pregrevica 118, 11080 Belgrade, Serbia. E-mail: krmpot@ipb.ac.rs

Supplemental Video S1 (description): Label-free THG 3D image reconstruction of live hypha

The 3D reconstruction of hyphae was made out of 23 THG images (slices) which are 0.9 μm apart with multiple acquisitions made at one point in repeated THG imaging (laser power 26 mW, microscope objective 40x 1.3 NA oil, wavelength 1040 nm, averaging 30, one image every 26 s). The VolView 3.4, open-source software, was used for making video of set of 2D images of hypha. The fungus was 23.5 hours old, grown in standard medium, then centrifuged and transferred to isoosmotic solution before THG imaging.

SUPPLEMENTARY MATERIALS AND METHODS

THG imaging conditions of lipid droplets

THG signal intensity strongly depends on local imaging conditions. Here, we describe key elements for development of lipid droplets THG imaging protocol:

- **The choice of objective:** (tested: THG imaging of live filamentous fungi using several types of objectives with different numerical apertures)
- **The choice of type of working liquid medium** (tested: THG imaging of live fungi in a standard liquid minimal (SLM) medium (glucose, salts, asparagine (amino acid), thiamine (vitamin), citric acid) vs isoosmotic extracellular solution (salts and sorbitol))

When the physiological objective, 40x 1.0 (**Figure S1a**), which has a relatively large working distance (2.5 mm), is inserted into a solution full of fungi (fungi in SLM medium), small portion of the intensity reaches the focal plane, the scattering is large and the focus is broken, which is expected for thick samples. Once we took the same fungi suspension that we hadn't seen lipid droplets on previously, and put them between two coverslips (fungi cells were sandwiched by two coverslips, 0.17 mm thickness) we could see prominent lipid droplets with 40x 1.3 oil immersion objective (**Figure S1c**). The thickness of the sample prepared in aforementioned way is 20 – 30 μm, while in chamber its thickness is ca 3 mm. The lipid droplet THG signal can be seen with 20x 0.8 air objective, but only with the increased power (**Figure S1b**).

When the fungi cells were centrifuged and resuspended in isoosmotic extracellular solution (**Figure S2**), it was possible to see lipid droplets with a physiological objective, 40x 1.0 (**Figure S2b**). The resulting image quality is close to that with an oil immersion objective, 40x 1.3, and hyphae sandwiched between two coverslips (**Figure S2a**). In this case, a lower cell density and a smaller number of large molecules in imaging “coverslip sandwich”, with subsequent reduction of the absorption of THG signals by cell membranes and large molecules, allows the visualization of lipid droplets in a deeper sample.

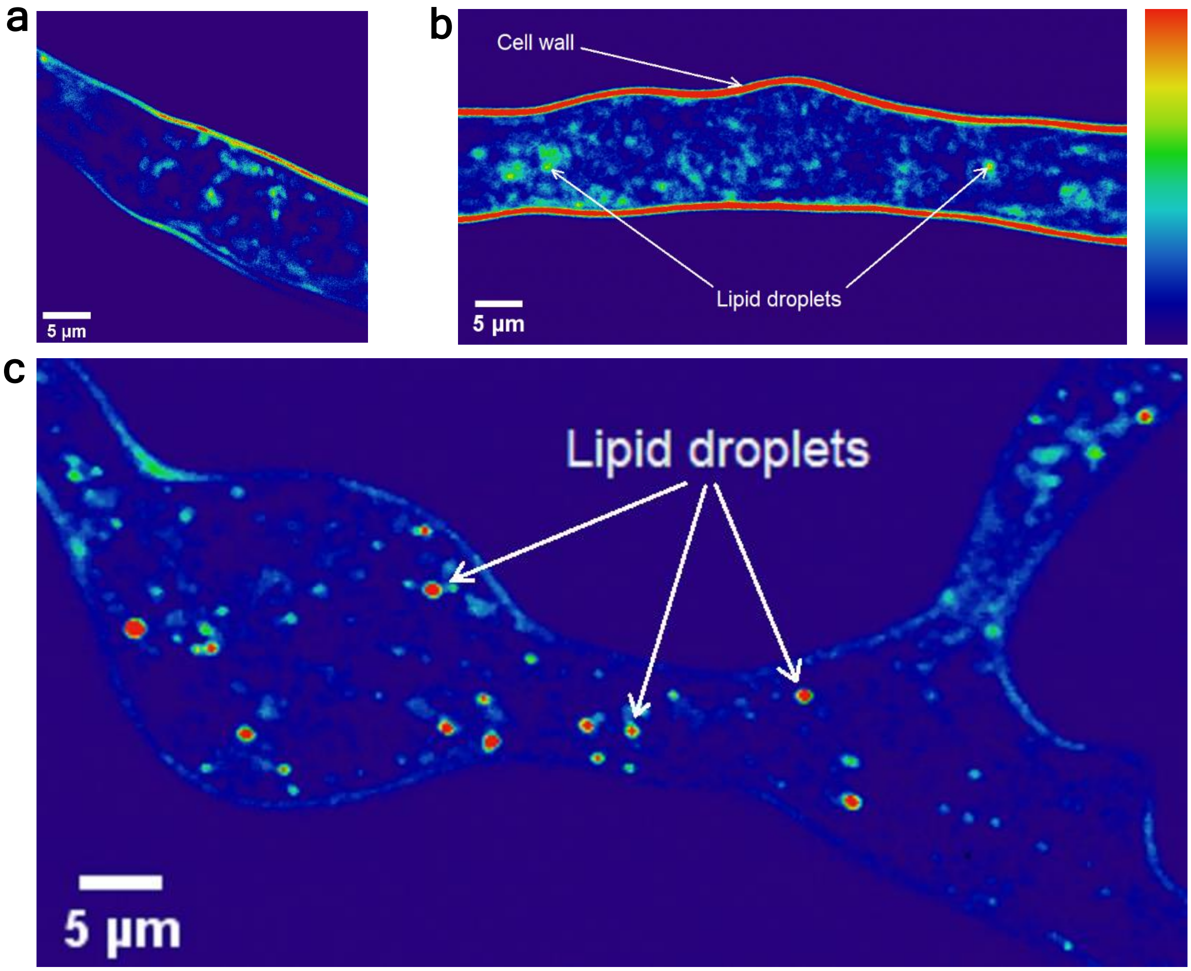

Supplementary Figure S1. Hyphae in SLM medium. (**a**) In the chamber (40x 1.0 physiological; laser power 58 mW), (**b**) Between two cover slips (20x 0.8; laser power 51 mW), (**c**) Between two cover slips (40x 1.3 oil; laser power 23 mW). Color intensity bar for THG signal: deep blue – the lowest signal, red the highest signal.

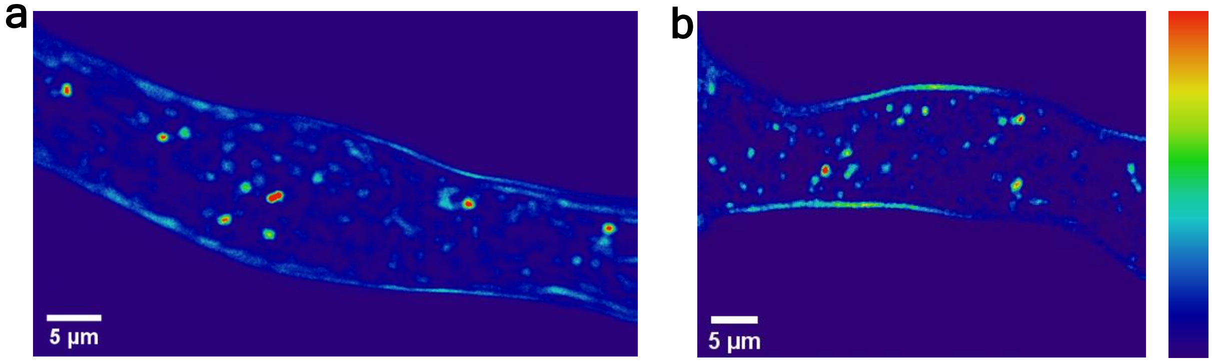

Supplementary Figure S2. Centrifuged hyphae in isoosmotic solution. (**a**) between two coverslips (34 mW laser power, oil immersion objective 40x 1.3) (**b**) in the chamber (39 mW, physiological objective 40x 1.0)

Dependence of THG signal on the laser power

Figure S3 shows the dependence of the intensity of the THG signal on the laser power. The small ROI (a part of the hypha), where the signal/noise ratio was good was taken for calculating the mean value of the THG intensity signal for various values of laser power. The graph clearly shows the third degree of dependence of the signal on the applied laser power.

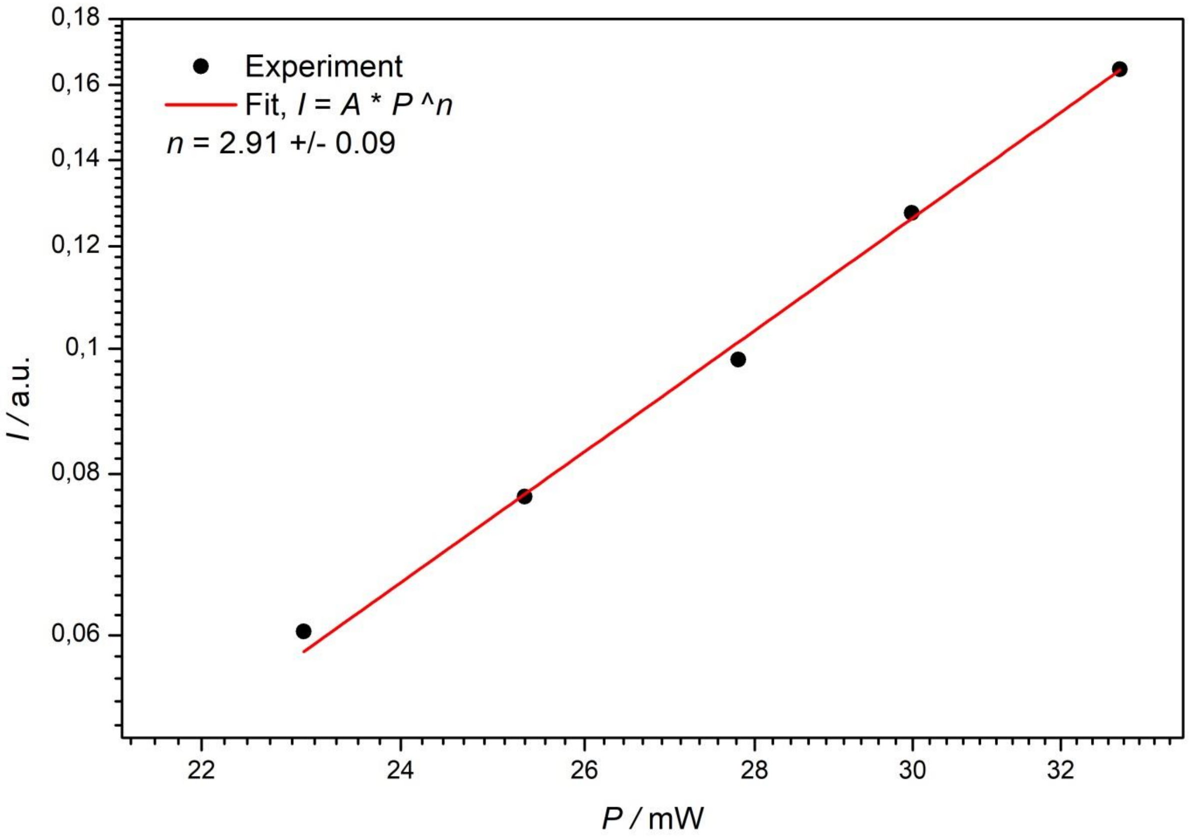

Supplementary Figure S3. Dependence of the THG signal intensity (*I*) on the laser power (*P*). The power degree obtained from the fit is n=2.91 ± 0.09. The specimen (in SLM medium) was placed between two coverslips.

The details of the image analysis

The details of the PSA procedure. Images were spatially scaled and thresholded. *Max Entropy* (automatic thresholding method (https://imagej.nih.gov/ij/plugins/entropy.html)) was applied. The threshold value for all images was between 0.03 and 0.1 percent. Afterwards, each image was converted to 8-bit mask which was used to derive lipid droplet data using the *Analyze Particles* tool. *Circularity* was limited to an interval of values from 0.3 to 1. *Circularity* ($4\pi \times$ [Area]/[Perimeter]^2, ranges from 0 (infinitely elongated polygon) to 1 (perfect circle). Minimum size limit was set to 0.3 µm. The *number*, *area* of lipid droplets, and *Feret's diameter* were calculated. *Feret's diameter* is the longest distance between any two points along the selection boundary.

The details of the ICS procedure. The average value of the background intensity was calculated as an average value of pixels in a small region of interest (ROI) that is outside of the hyphae. Then, the intensity of the background was subtracted from the 1024 x 1024 image 1, 2, 3, or 20 times and then saved in PNG format, closed and reopened in ImageJ. Autocorrelation calculation was done using the Menu Structure: *Process* > *FTT* > *FD Math* (Fourier techniques). The resulting image was normalized by dividing it by the total number of pixels and the square of the average intensity of the original area. The intensity profile was displayed after drawing a line through the center of the resulting ICS image and the plot was analyzed.

The details related to figure7

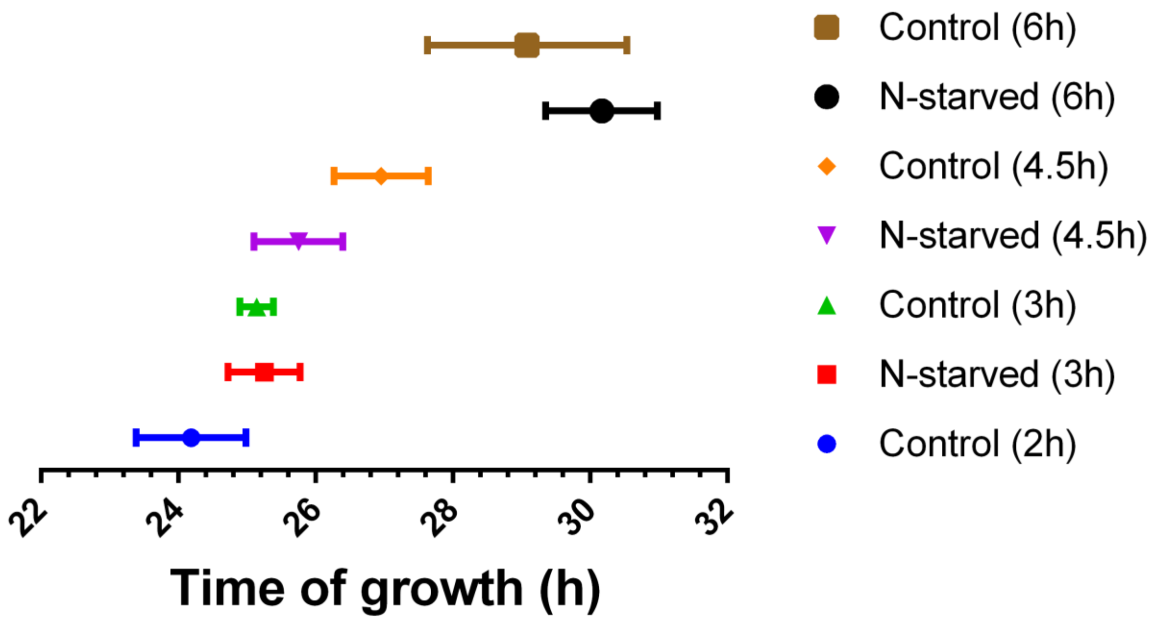

Supplementary Figure S4. The time errors for experimental groups depicted in Fig.7. Exact age of all hypha at the moment of imaging for each experimental group was pooled (mean ± SD).
